# Hydrogen cyanide produced by the soil bacterium *Chromobacterium* sp. Panama contributes to mortality in *Anopheles gambiae* mosquito larvae

**DOI:** 10.1038/s41598-018-26680-2

**Published:** 2018-05-29

**Authors:** Sarah M. Short, Sarah van Tol, Hannah J. MacLeod, George Dimopoulos

**Affiliations:** 1 W. Harry Feinstone Department of Molecular Microbiology and Immunology, Bloomberg School of Public Health, Johns Hopkins University, Baltimore, Maryland USA; 2 Present Address: Microbiology and Immunology, University of Texas Medical Branch, Galveston, Texas USA

## Abstract

Mosquito larvae continuously encounter microbes in their aquatic environment, which serve as food and play a critical role in successful development. In previous work, we isolated a *Chromobacterium* sp. (*C*.sp_P) with larvicidal activity from the midgut of dengue vector *Aedes* mosquitoes in Panama. In this study, we found a positive correlation between initial concentrations of *C*.sp_P and larval mortality rates, and that *C*.sp_P is more efficient at inducing larval mortality in a high nutrient environment. Multiple *Chromobacterium* species induce larval mortality with similar efficacy to *C*.sp_P except for *C*. *subtsugae*. We also found that a non-lethal dose of *C*.sp_P lengthens development time and increases mortality over multiple developmental stages, suggesting persistent effects of exposure. Additionally, we showed that larvicidal activity persists in the larval breeding water after removal of live bacteria, and that the larvicidal factor in *C*.sp_P-treated water is smaller than 3 kDa, heat resistant to 90 °C, and lost after vacuum centrifugation. We showed that *C*.sp_P produces hydrogen cyanide in culture and in larval water at concentrations sufficient to kill *An*. *gambiae* larvae, and treatment of the larval water with a cyanide antidote eliminated larvicidal activity. We conclude that a potential mechanism by which *C*.sp_P can induce larval mortality is via production of hydrogen cyanide.

## Introduction

Mosquitoes, including those transmitting human diseases, co-exist with diverse communities of bacteria throughout their lives, especially during the larval and pupal stages, when they dwell in bacteria rich bodies of water^1–4^. Bacteria serve as a food source for larvae, and colonization of the larval gut by bacteria is critical for proper larval development^5,6^. Recent work suggests that bacteria serve to reduce oxygen levels in the larval midgut which is a necessary trigger for metamorphosis^7^. The midgut microbiota of larvae is diverse and thought to be derived primarily from the larval habitat^2,4,5^. Its composition is commonly dominated by Proteobacteria, while Firmicutes, Bacteroidetes, Actinobacteria, and Cyanobacteria are also frequently detected in larvae^1,2,4,8^.

Bacteria in larvae can be transstadially transmitted to adult midguts^5^, though the mechanism of this transmission is unclear. This has implications for vector borne disease prevention, as the midgut microbiota of adult female mosquitoes has been shown to influence vector competence for *Plasmodium falciparum* (the etiological agent of malaria) and dengue virus^8,9^. For example, multiple bacteria prevent infection by *P*. *falciparum* when present in the midgut of adult female *An*. *gambiae*, a primary mosquito vector of this parasite^10–12^. Additionally, introduction of specific bacterial species to the midgut of *Ae*. *aegypti* (the primary vector of dengue) has been shown to decrease^12,13^ or increase^14^ mosquito susceptibility to dengue virus. Investigating and potentially exploiting interactions between environmental microbes and mosquito larvae can provide a better understanding of vector competence and presents novel opportunities for reducing vector borne disease transmission.

In addition to the potential influence on vector competence and vectorial capacity, study of mosquito-microbe interactions has the potential to yield novel biocontrol agents. The facts that larvae are filter feeders, are relatively stationary (compared to adults), and are confined to their breeding water render them good targets for microbial biocontrol^15^. The gram-positive sporulating bacteria *Bacillus thuringiensis* var *israelensis* was isolated from natural mosquito breeding sites and has been used in mosquito population control efforts successfully for decades, although mosquito resistance has been reported^16–18^. This and other species from this genus are ingested by mosquito larvae and release toxins in the larval digestive tract^16^. These toxins are primarily pore-forming proteins that cause lysis of cells in the insect gut resulting in eventual death^19–21^. Continued investigation of mosquito-associated bacteria in field populations has the potential to yield additional biocontrol agents.

In previous work, we found that *Chromobacterium* sp. Panama (*C*.sp_P), a bacterium isolated from the midguts of *Aedes aegypti* in Panama^13^, has larvicidal activity against *Anopheles gambiae* and *Ae*. *aegypti* larvae^12^. Other species of *Chromobacterium* have been shown to have insecticidal properties, including *C*.*vaccinii* which causes mortality in *A*. *aegypti* larvae as well as diamondback moths^22^. *C*. *subtsugae* induces mortality in multiple insect species, though not against larval *Culex pipiens* mosquitoes, and is the main ingredient in the biopesticide Grandevo® (Marrone BioInnovations)^23^. The most extensively studied member of the genus, *Chromobacterium violaceum*, produces multiple metabolites with medicinal and industrial applications, including violacin, which has antimicrobial and antiparasitic activities, and hydrogen cyanide (HCN), which can be used for bioleaching of gold from electronic waste and ore^24–27^. HCN is produced by hydrogen cyanide synthase encoded by the *hcnABC* operon which is present in *C*. *violaceum*, *C*. *vaccinii*, and multiple Pseudomonad genomes^22,25,28,29^. HCN production during *Pseudomonas* infection has been shown to contribute to lethality in *Caenorhabditis elegans* and *Drosophila melanogaster*^30,31^. *C*. *vaccinii* also produces HCN in culture, though it remains unclear whether HCN production induces mortality in larvae treated with these bacteria^22,29^. In the present study, we aimed to further characterize the larvicidal activity of *C*.sp_P and to better understand the mechanism of larval killing by the bacteria.

## Results

### Concentration of *C*.sp_P and larval diet influence larvicidal activity

**Figure 1:**
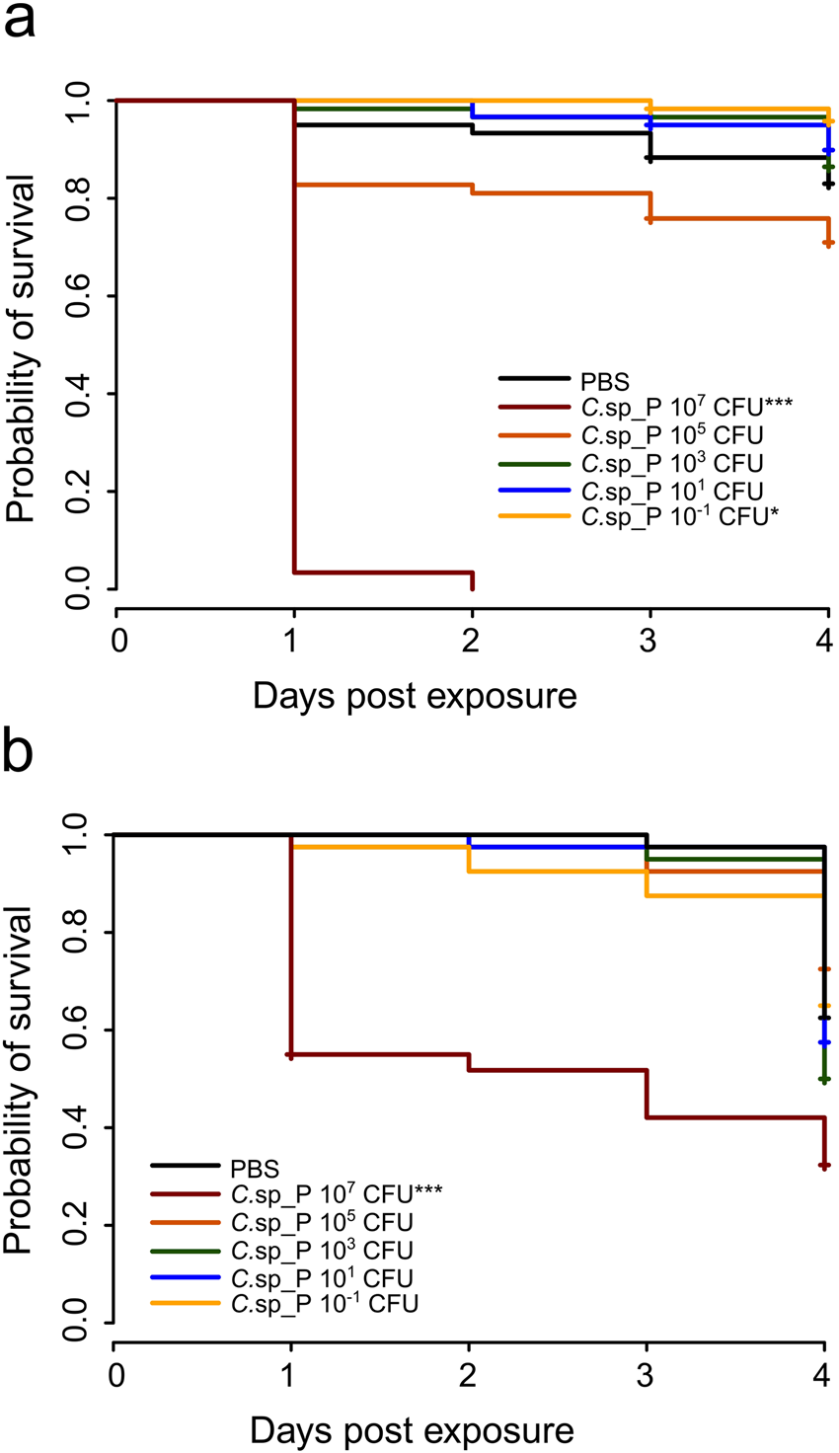
*C*.sp_P larvicidal activity depends on number of bacteria added to larval water as well as larval food availability. 1^st^ and 2^nd^ instar *An*. *gambiae* larvae were treated in groups of ten in 5 mL of water containing either 3 mg **(a)** or 1 mg **(b)** of ground larval food. *C*.sp_P overnight culture containing an average of 10^8^ CFUs was serially diluted and 100 µL of each dilution was added to the larval breeding water at concentrations of 10^7^, 10^5^, 10^3^, 10^1^ and 10^−1^ CFU per 5 mL larval water. 100 µL 1X PBS was added as a control and survival of larvae was monitored for four days. When 3 mg of larval food was present in the breeding water **(a)** addition of 10^7^ CFUs of *C*.sp_P resulted in significantly decreased survival compared to the PBS control (p < 0.001). Adding 10^−1^ CFU/5 mL resulted in a marginally significant increase in larval survival compared to PBS (p = 0.042). When 1 mg of larval food was present in the breeding water **(b)**, addition of 10^7^ CFUs of *C*.sp_P resulted in significant larval mortality compared to the PBS control (p < 0.001), and no other treatment influenced larval survival. Larval survival by day four for the 10^7^ CFU treatment was 0% and 32.4% when 3 mg and 1 mg of food were used, respectively. Each experiment was repeated three (**a**) or two (**b**) independent times and for each experimental replicate two pools of ten larvae were measured per treatment. Data were analyzed using a Cox proportional hazards model. Total sample sizes for (**a**) are as follows: PBS = 60, *C*.sp_P 10^7^ = 59, *C*.sp_P 10^5^ = 58, *C*.sp_P 10^3^ = 59, *C*.sp_P 10^1^ = 60, *C*.sp_P 10^−1^ = 59. Total sample sizes for (B) are as follows: PBS = 40, *C*.sp_P 10^7^ = 40, *C*.sp_P 10^5^ = 40, *C*.sp_P 10^3^ = 40, *C*.sp_P 10^1^ = 40, *C*.sp_P 10^−1^ = 40.

We investigated the conditions necessary to induce *An*. *gambiae* larval mortality by varying both the number of *C*.sp_P as well as the amount of larval food present in the larval water. To test the role of bacterial number, we added *C*.sp_P to larval breeding water at concentrations of 10^7^, 10^5^, 10^3^, 10^1^ and 10^−1^ Colony Forming Units (CFU) per 5 mL larval breeding water. To test the effect of larval food concentration, we conducted this experiment in water containing 3 mg ground larval food per 5 mL (“high nutrient,” Fig. 1b) and 1 mg ground larval food per 5 mL (“low nutrient,” Fig. 1b). We found that for both concentrations of larval food, adding 10^7^ *C*.sp_P was the only treatment that induced significant mortality (Fig. 1). By day four post exposure, probability of survival in low nutrient and high nutrient conditions was 32.4% and 0%, respectively. In addition, we showed that larval mortality is not induced by treatment with another bacterium isolated from the mosquito midgut, *Comamonas* sp., when added to larval breeding water at similar concentrations as *C*.sp_P (Fig. S1).

### Multiple species in the *Chromobacterium* genus induce larval mortality in *An*. *gambiae*

**Figure 2:**
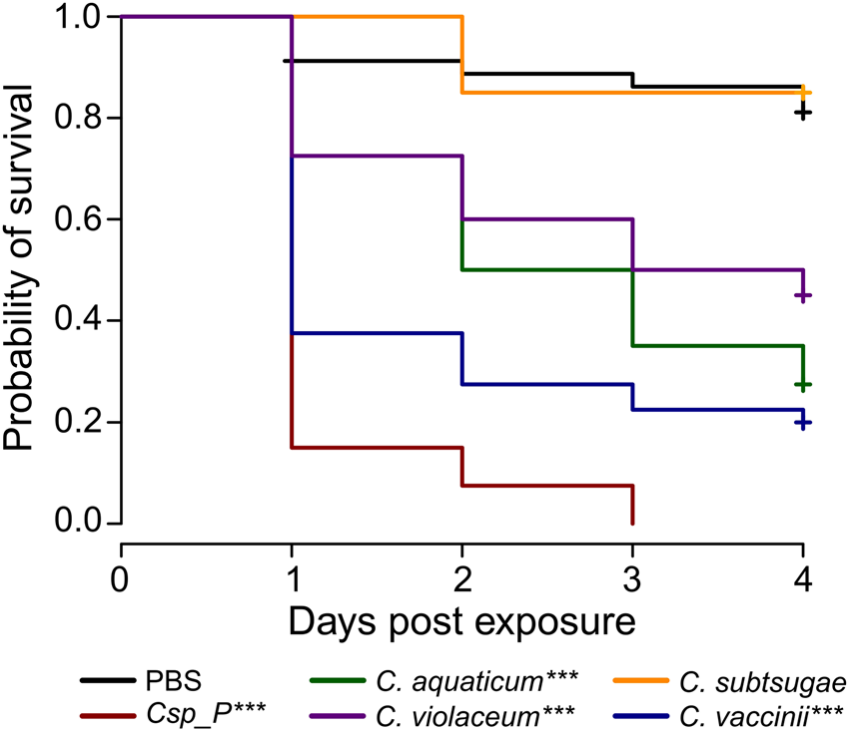
Multiple species of *Chromobacterium* have larvicidal activity. 1^st^ and 2^nd^ instar *An*. *gambiae* larvae were treated in groups of ten in 5 ml of distilled water containing 3 mg of ground larval food. *C*.sp_P, *C*. *aquaticum*, *C*. *violaceum*, *C*. *subtsugae*, and *C*. *vaccinii* were cultured overnight and washed twice with 1X PBS. *C*. *subtsugae* was further concentrated 5X and the other cultures were used without concentration. 100 µL of each culture or 1X PBS was added to the larval water and survival was monitored for four days. For all species except *C*. *subtsugae*, cultures contained an average of 10^8^ CFU/mL and the total number of bacteria added to the larval water was approximately 10^7^. *C*. *subtsugae* contained an average of 10^9^ CFU/mL, and the total number of bacteria added to the larval water was approximately10^8^. Compared to PBS, *C*.sp_P (p = 2 × 10^−16^), *C*. *aquaticum* (p = 5.46 × 10^−8^), *C*. *violaceum* (p = 4.63 × 10^−5^), and *C*. *vaccinii* (p = 3.66 × 10^−13^) showed significant larvicidal activity. *C*. *subtsugae* had no significant effect on larval survival (p = 0.846). Each treatment was repeated over a minimum of two independent experiments and for each experimental replicate one to two pools of ten larvae were measured per treatment. Data were analyzed using a Cox proportional hazards model. Total sample sizes are as follows: PBS = 80, *C*.sp_P = 40, *C*. *aquaticum* = 40, *C*. *violaceum* = 40, *C*. *subtsugae* = 20, *C*. *vaccinii* = 40.

We assessed whether other species related to *C*.sp_P were also capable of inducing larvicidal activity. For these assays, we chose *C*. *violaceum*, the most well-studied member of the genus, which produces multiple secondary metabolites with bioactive properties^24–26^. We also chose *C*. *subtsugae* which is the active ingredient in the bioinsecticide Grandevo® (Marrone BioInnovations)^23,32^, and *C*. *vaccinii* which has documented insecticidal activity against *Ae*. *aegypti* larvae^22^, though the insecticidal mechanisms of *C*. *subtsugae* and *C*. *vaccinii* remain unclear. We also used *C*. *aquaticum*, which does not produce violacein and is more closely related to *C*.sp_P than the other three species^12^. We found significant larval mortality over four days after treatment of larval breeding water with *C*.sp_P (p = 2 × 10^−16^), *C*. *aquaticum* (p = 5.46 × 10^−8^), *C*. *violaceum* (p = 4.63 × 10^−5^), and *C*. *vaccinii* (p = 3.66 × 10^−13^), but not *C*. *subtsugae* (p = 0.846) relative to the PBS control (Fig. 2). Bacterial concentrations of cultures used to treat larvae were highly similar between *C*.sp_P, *C*. *aquaticum*, and *C*.*vaccinii* (4 × 10^8^ CFU/mL on average), but higher for *C*. *violaceum* (8 × 10^8^ CFU/mL on average) and *C*. *subtsugae* (1 × 10^9^ CFU/mL on average) (Figure S2A). A regression analysis revealed no correlation between the CFUs added for each species and survival by day four (r^2^ = 0.01091, p = 0.2952, Figure S2B).

### Larvicidal activity persists after removal of live *C*.sp_P bacteria

**Figure 3:**
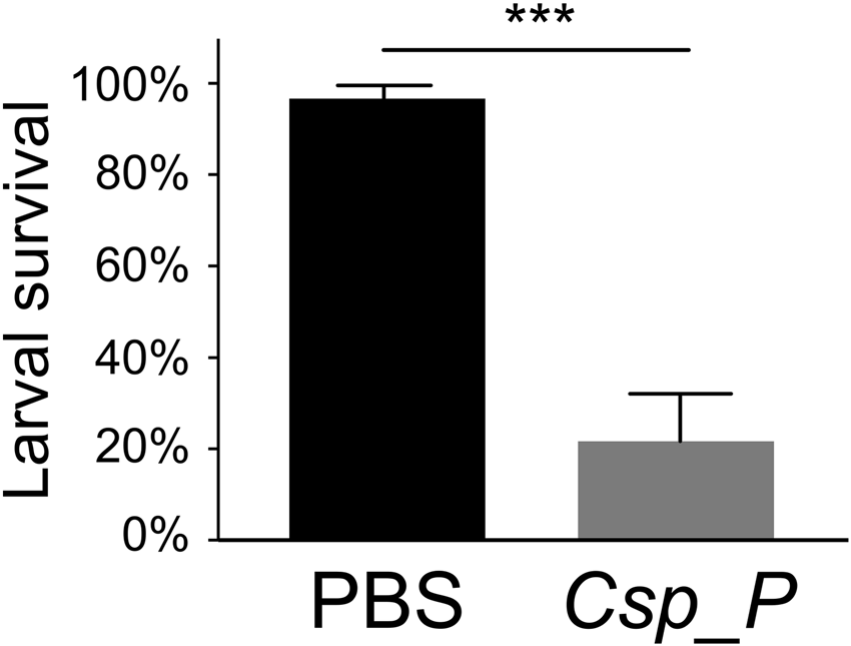
Larval water treated with *C*.sp_P retains larvicidal activity after removal of live bacteria. Larval breeding water was treated with either 1X PBS or *C*.sp_P, and incubated for 6–8 hours until all larvae in the *C*.sp_P treatment were dead. Larval water was then filtered using a 0.2 µm filter to remove all live bacterial cells. This filtered water was then used to treat new pools of ten *An*. *gambiae* larvae and survival was recorded after 16–17 hours exposure to the filtered water. Compared to the 1X PBS control, larvae exposed to filtered *C*.sp_P-treated water had significantly lower survival (p = 3.06 × 10^−5^). Data were analyzed using a binary logistic regression. The graph shows average percent survival across replicate experiments and error bars represent one standard deviation. For each experiment two pools of ten larvae were measured per treatment and the experiment was repeated three independent times (exception: experiment 1 has one pool of 10 per treatment). Sample size for both treatments was n = 50.

To determine whether live bacteria are necessary to induce larval mortality, we exposed *An*. *gambiae* larvae to *C*.sp_P-treated breeding water from which all live bacteria had been removed. To do this, we treated *An*. *gambiae* larval breeding water with *C*.sp_P until larval mortality was 100% and then passed the breeding water through a 0.2 µm filter. We then exposed additional *An*. *gambiae* larvae to the filtered breeding water and observed mortality after an overnight incubation. Larval mortality after exposure to filtered *C*.sp_P-treated breeding water was approximately 78%, while that in the PBS treated control was 3% (Fig. 3, p = 3.06 × 10^−5^).

We also investigated whether larvae incubated with *C*.sp_P could be contributing to the larvicidal activity in the filtered water (*e*.*g*. by releasing toxins after death). We repeated the experiment described above but excluded larvae from the initial breeding water. We observed significant mortality in the *C*.sp_P-treated water, similar to that seen when larvae were present in the initial incubation (Fig. S3, p = 0.00097). We were interested in determining the source of the larvicidal activity and began by first investigating the nature of the factor(s) causing larval mortality.

### *C*.sp_P larvicidal factor(s) are less than 3 kDa, heat stable, and volatile

**Figure 4:**
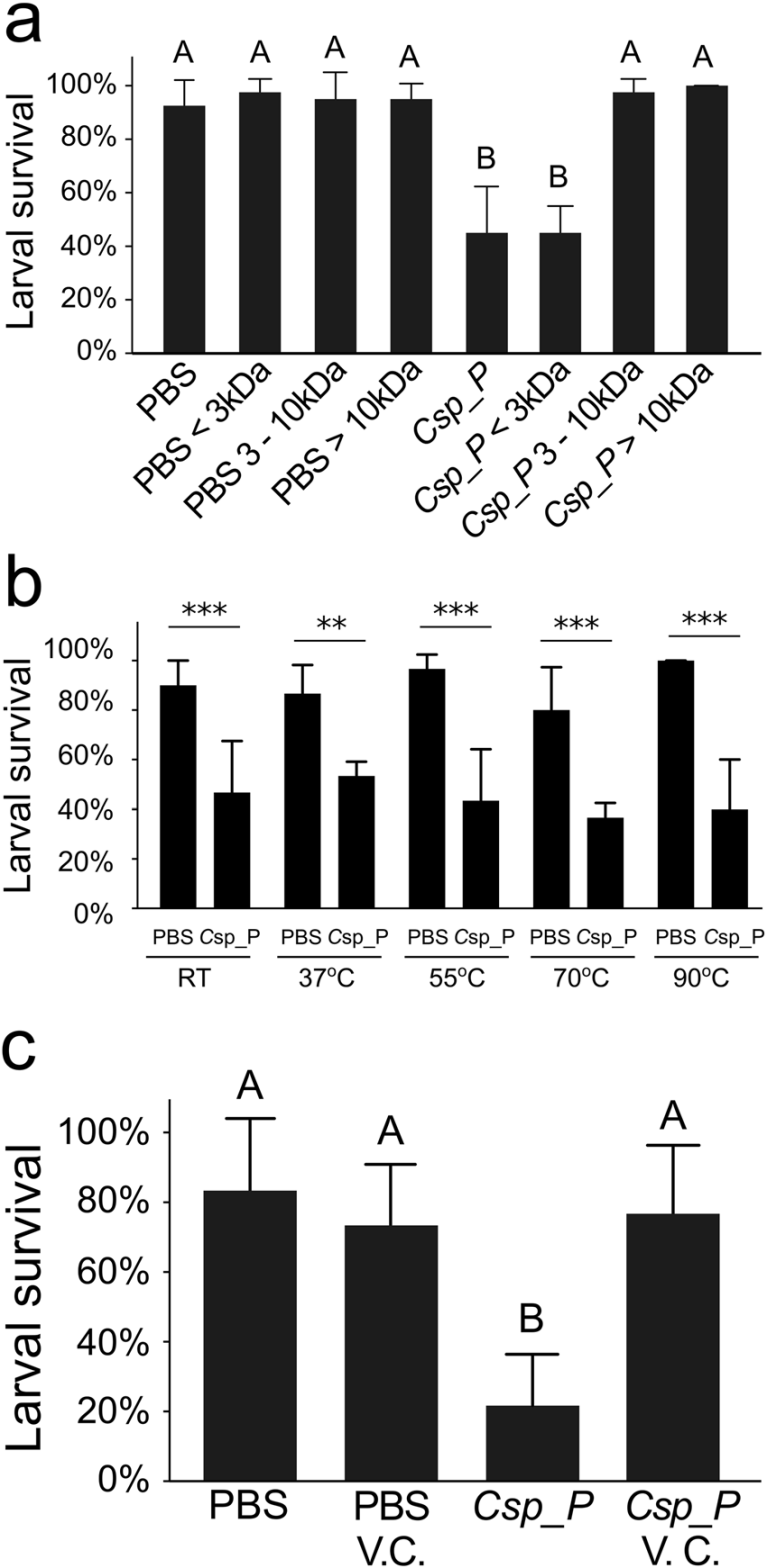
For all experiments, larval breeding water was treated with either 1X PBS or *C*.sp_P, incubated for 6–8 hours, collected and filtered using a 0.2 µm filter to remove all live bacterial cells. (**a**) *C*.sp_P produced larvicidal factor is less than 3 kDa. Filtered water was size-fractionated using centrifuge filtration and used to treat *An*. *gambiae* larvae. Survival was recorded 16–17 hours after exposure. Larvae exposed to unfractionated *C*.sp_P-treated water had significantly lower survival compared to unfractionated 1X PBS (p = 0.0011), and the *C*.sp_P < 3 kDa fraction had significantly more larval mortality compared to the 1X PBS < 3 kDa control (p = 0.0043). Data were analyzed using a binary logistic regression. (**b**) *C*.sp_P produced larvicidal factor is heat resistant. Filtered water was heated for one hour at the indicated temperatures. Each water sample was used to treat *An*. *gambiae* larvae and survival was recorded 16–17 hours after exposure. The effect of *C*.sp_P treatment was significant for all temperatures (room temperature (RT), p = 1.17 × 10^−4^; 37 °C, p = 0.00354; 55 °C, p = 8.28 × 10^−7^; 70 °C, p = 4.57 × 10^−4^; 90 °C, p = 4.39 × 10^−9^). Data were analyzed within each temperature using a binary logistic regression. Control data are a subset of those used in 4a. (**c**)Vacuum centrifugation eliminates larvicidal activity produced by *C*.sp_P. PBS and *C*.sp_P-treated filtered water samples from three replicates of the experiment shown in Panel (**a**) were vacuum centrifuged (V.C.) for 30 minutes and used to treat *An*. *gambiae* larvae. Survival was recorded after 24 hours. Data were analyzed using a binary logistic regression, and a two-way ANOVA showed a significant interaction between treatment (PBS vs. *C*.sp_P) and effect of vacuum centrifugation (p = 1.60 × 10^−7^). *C*.sp_P treatment resulted in significant larval mortality (p < 1.0 × 10^−4^), but this was not the case when the samples were vacuum centrifuged (p = 0.972). Graphs show average percent survival across replicate experiments and error bars represent one standard deviation. For (**a**,**b**), two pools of five larvae were measured per treatment and the experiment was repeated 4 and 3 independent times, respectively. For (**c**), two pools of five larvae were measured and the full experiment was repeated twice.

We size fractionated filtered (*i*.*e*. cell-free) *C*.sp_P- and PBS-treated larval water using centrifuge filtration to obtain three fractions: (1) molecules greater than 10 kDa, (2) molecules less than 10 kDa but more than 3 kDa, and (3) molecules less than 3 kDa. We then treated *An*. *gambiae* larvae with each fraction as well as the whole filtrate. We found that larvae treated with the whole *C*.sp_P filtrate and the <3 kDa fraction experienced similar levels of larval mortality which significantly differed from the respective PBS controls as well as the *C*.sp_P fractions containing larger molecules (Fig. 4a; *C*.sp_P_whole_ vs. PBS_whole_, p = 0.0011; *C*.sp_P_<3kDa_ vs. PBS_<3kDa_, p = 0.0043).

We also investigated the heat sensitivity of the *C*.sp_P-produced larvicidal factor. We filter sterilized *C*.sp_P- and PBS-treated larval breeding water, incubated the water for one hour at room temperature (RT), 37 °C, 55 °C, 70 °C, or 90 °C, and then treated *An*. *gambiae* larvae with each sample and observed mortality. We found that exposure to *C*.sp_P-treated water resulted in significant larval mortality relative to the respective PBS control for all treatments (Fig. 4b; *C*.sp_P_RT_ vs. PBS_RT_, p = 1.17 × 10^−4^; *C*.sp_P _37°_ vs. PBS_37°_, p = 0.00354; *C*.sp_P _55°_ vs. PBS_55°_, p = 8.28 × 10^−7^; *C*.sp_P _70°_ vs. PBS_70°_, p = 4.57 × 10^−4^; *C*.sp_P _90°_ vs. PBS_90°_, p = 4.39 × 10^−9^).

Finally, we investigated whether the larvicidal factor produced by *C*.sp_P is volatile. We centrifuged filtered *C*.sp_P- and PBS-treated larval breeding water in a vacuum centrifuge for 30 minutes to allow volatile compounds to evaporate. We then treated *An*. *gambiae* larvae with centrifuged and uncentrifuged water. Exposure to *C*.sp_P-treated water resulted in significant larval mortality in uncentrifuged samples (*C*.sp_P vs. PBS, p < 1.0 × 10^−4^), but not in centrifuged samples (Fig. 4c; *C*.sp_P _VC_ vs. PBS_VC_, p = 0.972).

### *C*.sp_P produces cyanide in breeding water at concentrations sufficient to induce larval mortality

**Figure 5:**
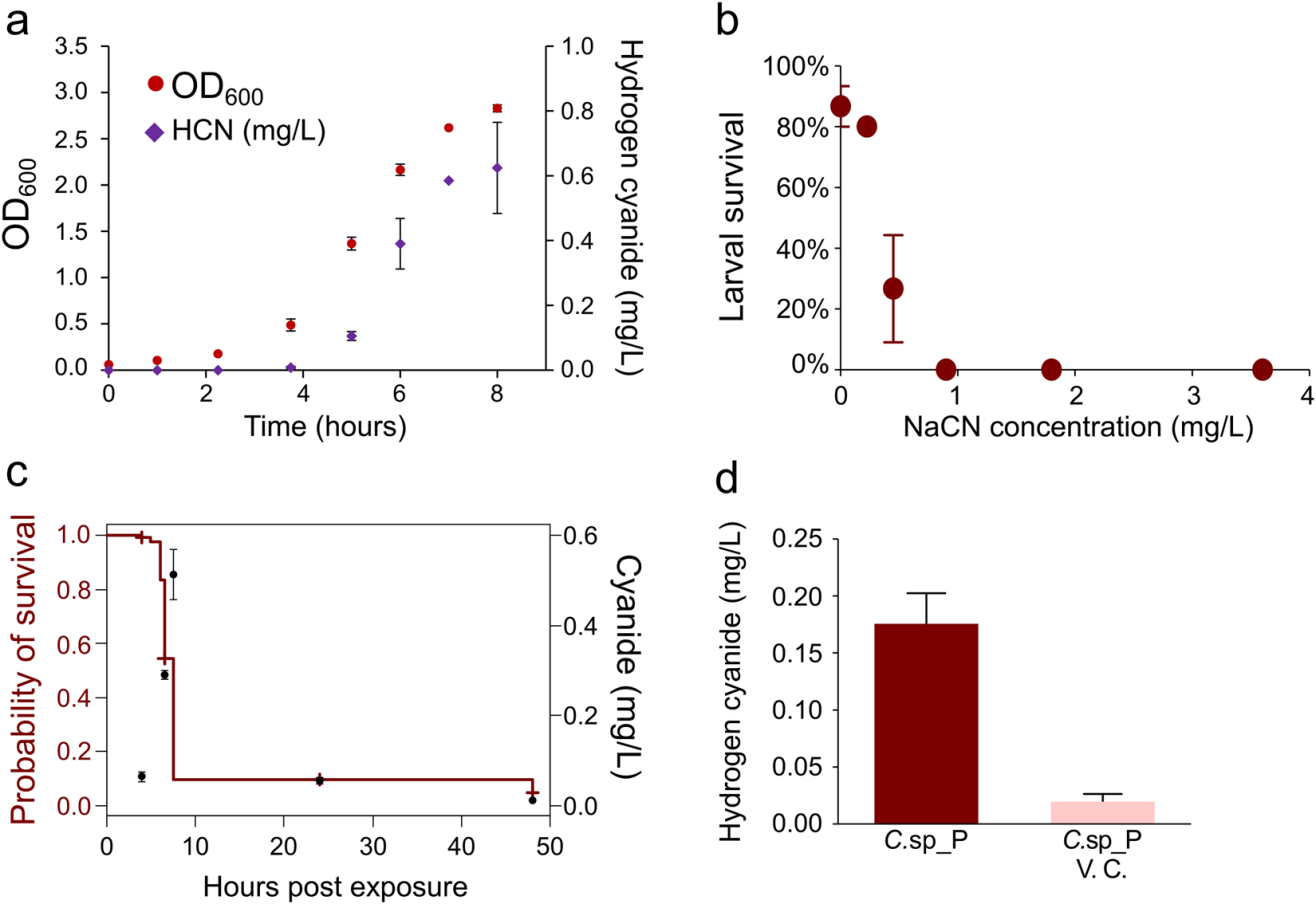
Cyanide production by *C*.sp_P in culture and larval breeding water. (**a**) *C*.sp_P produces hydrogen cyanide during log phase of bacterial growth. One liter of liquid LB was inoculated with 10 mL of an overnight *C*.sp_P culture and grown at 30 °C with shaking for eight hours. At 1-1.5 hour intervals, absorbance at 600 nm was measured as well as hydrogen cyanide concentration using the Hach Cyanide Test Kit CYN-3 (Lot A5316). Three cultures were measured in triplicate, and error bars represent one standard error. (**b**) Assessment of environmental cyanide concentration required to induce *An*. *gambiae* larval mortality. An 18 mg/mL stock solution of NaCN dissolved in water was serially diluted 1:5, 1:10, 1:20, 1:40, and 1:80 and each dilution (plus a water control) was used to treat three pools of five *An*. *gambiae* larvae. Survival for each treatment was recorded 24 hours after initial exposure. Larval mortality is substantial but variable in pools treated with 0.45 mg/L (1:40 dilution), and total mortality is induced at 0.9 mg/L (1:20 dilution). Sample size for each treatment was n = 15. (**c**) *C*.sp_P-induced larval mortality co-occurs with increased cyanide concentration. Pools of ten *An*. *gambiae* larvae in 75 mL breeding water were treated with 500 µL 1.0 OD_600_ *C*.sp_P bacterial culture. Survival was recorded at 3, 4, 5, 6, 6.5, 7.5, 24, and 48 hours after treatment, and cyanide concentration was measured at 4, 6.5, 7.5, 24, and 48 hours after treatment. Semi-automated colorimetry (EPA Method 335.4) was used to measure cyanide in triplicate at each time point. Survival is shown in red while cyanide concentration is shown in black. (**d**) Vacuum centrifugation eliminates hydrogen cyanide from *C*.sp_P-treated water. Larval breeding water was treated with *C*.sp_P, and incubated for approximately 7 hours. Larval water was then collected and filtered using a 0.2 µm filter to remove all live bacterial cells. Aliquots of the filtered water samples were vacuum centrifuged for 30 minutes at room temperature and HCN was quantified using the Hach Cyanide Test Kit CYN-3 (Lot A5316) in centrifuged and control samples. The experiment was performed in triplicate, and error bars represent one standard deviation.

Hydrogen cyanide, known to be produced by multiple *Chromobacterium* species^22,33^, is a volatile and heat stable small molecule^34,35^ and therefore could be the source of *C*.sp_P’s larvicidal activity. We first determined whether *C*.sp_P produces HCN in culture and the dynamics of its production during growth. We found that cyanide levels began to increase during the log phase of *C*.sp_P growth and reached approximately 0.6 mg/L by the beginning of stationary phase (Fig. 5a).

We next assessed the concentration of cyanide in the aquatic environment that would be necessary to cause larval mortality. We submerged *An*. *gambiae* larvae for 24 hours in a dilution series of NaCN solution, with concentrations ranging from 0 mg/L to 3.6 mg/L. We found that larval mortality was similar to the negative control (approximately 20% mortality) in the 0.23 mg/L treatment but increased to 73.3% in the 0.45 mg/L treatment and was 100% in the 0.9 mg/L treatment (Fig. 5b).

Additionally, we assessed the level of HCN produced by *C*.sp_P in larval breeding water and the extent to which its production correlates with larval mortality (Fig. 5c). At 4 h post exposure, average cyanide concentrations in larval breeding water were 0.063 mg/L and larval mortality was 0.7%. At 6.5 h post exposure, larval mortality was 45.7% and average cyanide concentrations 0.29 mg/L. By 7.5 h post exposure, larval mortality was 90.6% and average cyanide concentrations had increased to 0.513 mg/L. By 24 h and 48 h, average cyanide concentrations decreased to 0.055 mg/L and less than 0.01 mg/L, respectively.

Finally, we investigated whether HCN evaporates from *C*.sp_P-treated larval water when centrifuged under a vacuum. Vacuum centrifugation eliminated larvicidal activity (Fig. 4c); if HCN is causing larval mortality, we predicted it would no longer be present after vacuum centrifugation. We found that after vacuum centrifugation, nearly all HCN was lost, with HCN levels decreasing nearly ten-fold (Fig. 5d, *C*.sp_P HCN = 0.175 mg/L, *C*.sp_P_VC_, HCN = 0.02 mg/L).

### Treatment with the cyanide antidote hydroxocobalamin eliminates *C*.sp_P larvicidal activity

**Figure 6:**
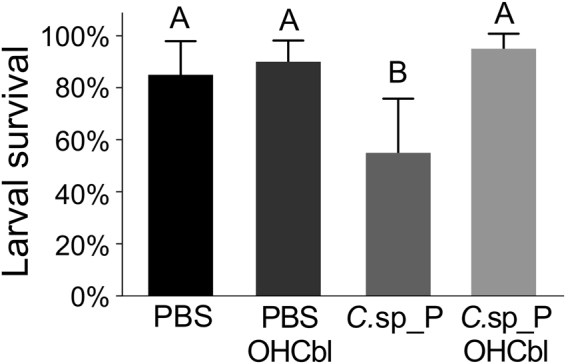
Treatment with cyanide antidote OHCbl eliminates larvicidal activity from *C*.sp_P-treated water. *C*.sp_P- or PBS-treated water was filtered (*i*.*e*. made cell-free) and used to treat pools of *An*. *gambiae* larvae. To half of the pools, OHCbl was added at a final concentration of 0.2 mg/ml. Survival was recorded after 16–17 hours exposure to the filtered water. Data were analyzed using a binary logistic regression, and a two-way Analysis of Variance showed a significant interaction between water treatment (PBS vs. *C*.sp_P) and effect of adding OHCbl (p = 0.0245). Pairwise comparisons revealed that larval survival was significantly lower in *C*.sp_P-treated water compared to the 1X PBS control (p = 0.0242), but this was not the case when the samples were treated with OHCbl (PBS/OHCbl vs. *C*.sp_P/OHCbl p = 0.834). Breeding water was collected on two independent days and was used to treat larvae in two replicate experiments. The graph shows average percent survival across all experiments and error bars represent one standard deviation. For each experiment, two pools of five larvae were used for each treatment, and for all treatments, sample size was n = 40. OHCbl = Hydroxocobalamin.

We supplemented filtered (*i*.*e*. cell-free) *C*.sp_P-treated larval breeding water with hydroxocobalamin (OHCbl), which sequesters cyanide and is used as an antidote for cyanide poisoning^36^. We found significant mortality in larvae exposed to *C*.sp_P-treated water (Fig. 6, *C*.sp_P vs. PBS, p = 0.024), but no effect on larval survival when *C*.sp_P-treated water was supplemented with OHCbl (Fig. 6, *C*.sp_P_OHCbl_ vs PBS_OHCbl_, p = 0.834).

### Surviving initial exposure to *C*.sp_P slows development and reduces survival over time

**Figure 7:**
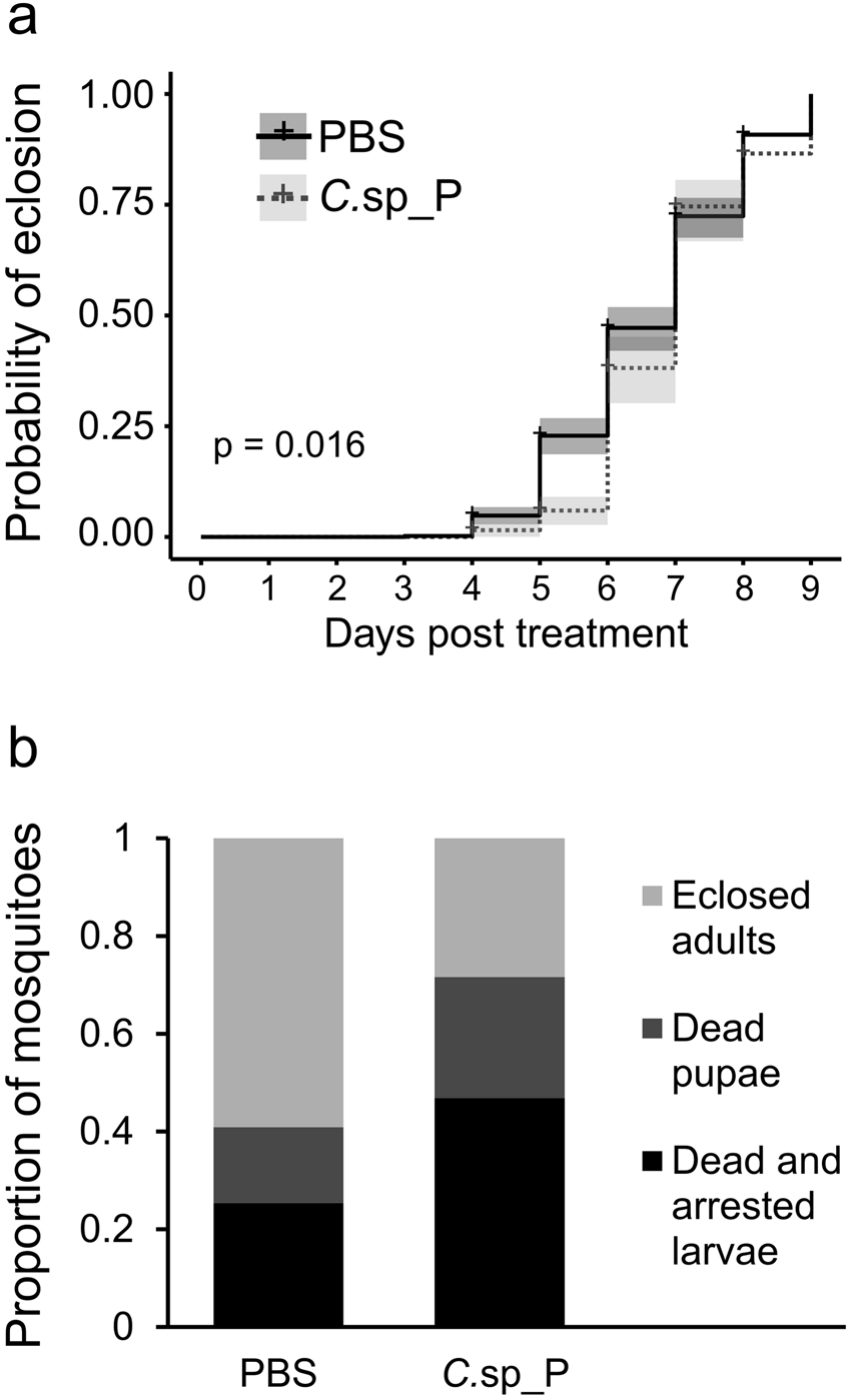
Short term non-lethal exposure of larvae to *C*.sp_P slows eclosion and reduces probability of survival to adulthood. *An*. *gambiae* larvae were treated with *C*.sp_P or 1X PBS for two days in groups of 350 at which point larval survival was recorded (mean survival was 91.1% in PBS and 70.4% in *C*.sp_P) and larvae were rinsed twice with distilled water and transferred to clean larval rearing pans. Pupation and eclosion events were recorded daily until all pupae eclosed or died. The total number of larvae that either died as larvae or failed to pupate (arrested) by day 11 post treatment was also recorded. The entire experiment was repeated twice. **(a**) Rate of eclosion is slower in *C*.sp_P-exposed individuals. Among individuals that successfully pupated, those exposed to *C*.sp_P had a significantly slower rate of eclosion over time relative to PBS controls (*C*.sp_P vs. PBS, p = 0.016). Probability of eclosion over time was analyzed by log rank test, and pupal deaths were treated as censored data. **(b**) Overall probability of pupation and eclosion is lower in *C*.sp_P-exposed individuals. When larvae were treated with *C*.sp_P, a lower proportion successfully pupated by nine days post treatment (total pupation for *C*.sp_P_rep1_ = 45.5%, *C*.sp_P_rep2_ = 59.4%, PBS_rep1_ = 64.7%, PBS_rep2_ = 82.8%). Of those that pupated, *C*.sp_P-treated individuals were less likely to eclose (total eclosion for *C*.sp_P_rep1_ = 47.5%, *C*.sp_P_rep2_ = 57.1%, PBS_rep1_ = 62.0%, PBS_rep2_ = 90.3%). The figure shows overall proportions from pooled data from two experimental replicates.

We were interested in determining whether there are persistent effects of non-lethal exposure to *C*.sp_P on mosquito survival and/or fitness. We exposed *An*. *gambiae* larvae to a moderate dose of *C*.sp_P (or PBS as a control) for two days. After two days, we recorded larval survival, which averaged 91.1% in PBS and 70.4% in *C*.sp_P, rinsed larvae from both treatments in clean distilled water and transferred larvae to clean trays containing additional distilled water. We then monitored pupation events, pupal death, and eclosion events until all pupae had either eclosed or died. We also recorded the total number of larvae that either failed to pupate by 11 days or died as larvae. We found that early exposure to *C*.sp_P significantly slowed eclosion (Fig. 7a, *C*.sp_P vs. PBS, p = 0.016). This effect was strongest at five days after transfer to clean water, and by day seven the probability of eclosion was similar between the two treatments. We also found that when larvae were treated with *C*.sp_P, by the end of the experiment a lower proportion had successfully pupated (Fig. 7b; overall percent pupation for *C*.sp_P = 53.1%, PBS = 74.6%), and of those that pupated, *C*.sp_P-treated individuals were less likely to eclose (Fig. 7b; overall percent of pupae that eclosed: *C*.sp_P 53.4% PBS, 79.2%).

## Discussion

In the current work, we aimed to characterize the larvicidal activity of *C*.sp_P and determine potential mechanism(s) by which *C*.sp_P causes mosquito larval mortality when present in the larval breeding water. We found that larvicidal activity is retained after removal of live *C*.sp_P from breeding water, indicating that the bacteria are producing a factor or factors that act(s) independently of live bacteria. This activity is derived primarily from *C*.sp_P and not from the larval carcasses, as larvicidal activity was present in breeding water that contained *C*.sp_P and no larvae. The larvicidal factor(s) is/are less than 3 kDa in size, potentially suggesting a short peptide or small molecule. Activity persisted after heat treatment as high as 90 °C for one hour, and it was lost after vacuum centrifuging the breeding water for 30 minutes. We also found that *C*.sp_P produced hydrogen cyanide in larval breeding water at concentrations sufficient to result in larval mortality, and vacuum centrifugation of *C*.sp_P-treated larval breeding water resulted in near elimination of HCN from the water and a concurrent loss of larvicidal activity. Treatment with hydroxocobalamin (OHCbl), a cyanide antidote, also eliminated larvicidal activity from *C*.sp_P-treated breeding water. Taken together, these results indicate that larval mortality is at least in part a result of HCN accumulation in the larval breeding water, eventually reaching environmental levels that are toxic to larvae and then gradually evaporating over time. It is not clear from these data, however, that HCN is the only source of larvicidal activity produced by *C*.sp_P or what other factors may also be involved.

The fact that exposure to cell-free *C*.sp_P-treated water resulted in larval mortality is consistent with the hypothesis that HCN is a source of larvicidal activity, as HCN synthase is a membrane bound protein and cyanide accumulates in the supernatant during bacterial growth^28,33^. HCN has also been shown to be heat resistant above 90 °C and is volatile^34,35^, suggesting that it would persist in breeding water after heat treatment and would be lost after vacuum centrifugation. OHCbl acts by binding cyanide directly to form non-toxic cyanocobalamin, also known as vitamin B12^36^. Accordingly, we show that addition of an excess of OHCbl to the filtered *C*.sp_P-treated larval breeding water abolished larvicidal activity. OHCbl can bind a variety of other molecules besides HCN, including many small anionic molecules (e.g. nitric oxide and sulfite) as well as larger molecules like glutathione and other thiols^37–40^, so we cannot rule out the possibility that OHCbl may have bound other molecules that contribute to larval mortality. However, OHCbl has a high affinity for HCN^37^ suggesting it would readily bind cyanide in the larval water, and the results of this experiment as well as the additional findings in the manuscript are consistent with the hypothesis that HCN production by *C*.sp_P is a source of larval mortality.

HCN production by *Pseuodmonas aeruginosa* has been shown to cause mortality in *Caenorhabditis elegans*, and to also play a role in virulence against *Drosophila melanogaster*^30,31^. To our knowledge, this is the first evidence of bacteria-produced HCN causing mortality in a mosquito.

Larvicidal activity was persistently lower in filtered (*i*.*e*. bacteria-free) *C*.sp_P-treated water relative to larval water containing live *C*.sp_P. It is possible that when live *C*.sp_P are in the breeding water it contains more HCN over longer periods of time, whereas HCN is expected to be continually evaporating from filtered water during exposure, resulting in gradually reduced levels over time. It is also possible that live *C*.sp_P are ingested by the larvae, and that internal exposure to the bacteria contributes to mortality, either through HCN production or an alternative mechanism. We note that hypoxia has been reported in the larval gut, which is a necessary environmental trigger for HCN production^7,41^. A third possibility is that *C*.sp_P produces additional factors that are not maintained in filtered preparations that either act to compound the effects of HCN or to induce larvicidal activity through an unrelated mechanism. Further investigation into whether *C*.sp_P produces larvicidal compounds in addition to HCN is warranted.

We found that larval mortality induced by *C*.sp_P was dependent on larval diet concentration. In a “low nutrient” environment (1 mg diet/5 ml water) vs. “high nutrient” (3 mg diet/5 ml water), the larvicidal activity of *C*.sp_P was substantially reduced. *C*. *violaceum*, *C*. *vaccinii*, and several species in the genus *Pseudomonas* have been observed to produce HCN and this process is highly influenced by nutritional availability^22,33,42–44^. HCN is a secondary metabolite produced as a byproduct of glycine metabolism, and is therefore highly dependent on glycine availability^33,45–47^. While glycine is the precursor of HCN formation, other amino acids influence HCN production, such as threonine which can be converted to glycine, and methionine which synergistically enhances the effect of glycine availability on HCN formation^33,45^. HCN production is also influenced by environmental levels of iron. Increased concentrations of iron in growth media causes higher HCN production in *C*. *violaceum* and *P*. *aeruginosa*^42,48^. Given the multiple nutritional requirements for HCN production, the “low nutrient” condition may not have contained enough of one of the molecules mentioned above to support lethal levels of HCN production.

In *Pseudomonas*, HCN production has also been shown to be regulated by quorum sensing^49^ and oxygen availability; microaerophilic (but not anaerobic) conditions promote HCN production through regulation of oxygen sensitive transcription factor complexes^28,41^. HCN production is therefore highest in late log phase, after a period of rapid oxygen consumption and when high cell densities enable quorum sensing^49^. We found this to also be the case for *C*.sp_P; the highest concentration of HCN in culture was during late log phase. In larval breeding water, in a “high nutrient” environment over the course of four days, *C*.sp_P induced little mortality when added at 1 × 10^5^ CFU/5 mL, but 100% mortality when added at 1 × 10^7^ CFU/5 mL. HCN production would not be predicted to occur at low bacterial densities, and the fact that mortality did not occur by four days in the treatments with fewer initial bacteria suggests that the bacteria could not replicate enough in the breeding water to reach densities sufficient to trigger quorum sensing. In a “low nutrient” environment, even addition of 1 × 10^7^ CFU/5 mL was not sufficient to induce 100% mortality. As discussed above, this may be due to a lack of nutrients essential for HCN production. Alternatively, it is possible that reduced nutrient availability may slow metabolic activity of *C*.sp_P, preventing bacterial replication and/or oxygen consumption from reaching levels high enough to induce HCN production.

We found that exposure to *C*. *violaceum*, *C*. *aquaticum*, and *C*. *vaccinii* also resulted in larval mortality, suggesting the effect is widespread throughout the *Chromobacterium* genus. *C*. *subtsugae* did not have any observable larvicidal activity, despite being added to larval breeding water at higher concentrations than the other species. These findings are consistent with previous work showing that *C*. *vaccinii* has larvicidal activity against *A*. *aegypti* but *C*. *subtsuage* does not against *C*. *pipiens*^22,23^. It has been previously shown that *C*. *violaceum* produces HCN in culture, as does *C*. *vaccinii*.^22,29,33^, and this may be the mechanism by which these species cause larval mortality. *C*. *subtsugae* does not produce HCN above background levels when cultured in King’s Medium B, suggesting that this species does not produce HCN even under optimal growth conditions^22^. It remains unclear why *C*. *subtsugae* fails to produce HCN, though we speculate that its lack of HCN production may underlie its inability to induce mortality in mosquito larvae.

Cyanide binds to cytochrome c oxidase, the final enzyme in the electron transport chain, and inhibits cellular respiration, leading to anoxia and cell death^50^. The nervous system is particularly susceptible to cyanide poisoning and collapse of nervous system function leads to subsequent respiratory system failure and death^51^. Because cyanide acts on such a common and critical cellular process, it is a broad-spectrum toxin, capable of inducing mortality in organisms ranging from bacteria to mammals. Cyanide’s mode of action may also greatly limit the mosquito’s ability to evolve resistance to the poison. Cyanide resistant respiration is not uncommon in microorganisms and plants, but it is rare among animals^52–54^. Notably, however, there is at least one instance of cyanide resistance arising in a scale insect population that was regularly treated with hydrogen cyanide^55^, and some species of herbivorous insects have evolved the ability to tolerate ingestion of cyanogenic plants^56–58^. Cyanide induces mortality in aquatic invertebrates at concentrations less than 100 µg/L^59^. In fish, mortality is induced at environmental cyanide concentrations as low as 24 µg/L in Atlantic salmon and as low as 57 µg/L in rainbow trout^59,60^. Cyanide is rapidly detoxified and does not bioaccumulate, but sub-lethal doses can have long-term effects on reproduction and development in freshwater fish^51^. In humans, sublethal doses through consumption of cyanogenic foods such as cassava can cause multiple health problems such as spastic paraparesis and thyroid disease^61–64^. The EPA’s recommended maximum contamination level for HCN in drinking water is 200 µg/L and according to the WHO a tolerable daily intake for adults is 12 µg/kg of body weight^65,66^. At peak HCN production, consumption of 1.5 L of *C*.sp_P-treated water by a 70 kg individual would exceed this recommended daily intake limit.

Sub-lethal exposure of *An*. *gambaie* larvae to *C*.sp_P results in significant delays in the rate of eclosion and reduced survival over time, with increased mortality observed in larval and pupal stages and a lower overall proportion of eclosing adults. HCN produced by *C*.sp_P during the sub-lethal exposure may cause delayed mortality, or it may be due to another factor produced by the bacteria during this time. Another, non-mutually exclusive explanation is that *C*.sp_P may colonize the larval midgut after initial exposure, and chronic internal exposure to the bacteria over time could lead to delayed mortality and slowed development time. Regardless of the mechanism, our study suggests that *C*.sp_P could have long-term negative effects on fitness of vector mosquitoes even when given at a sub-lethal dose.

In conclusion, we showed that *C*.sp_P is a cyanogenic bacterium that induces death in larval *An*. *gambiae* mosquitoes at least in part via production of hydrogen cyanide in larval breeding water. At sub-lethal doses, it also results in reduced fitness potential in *An*. *gambiae*. We also found that larvicidal activity is widespread throughout the *Chromobacterium* genus; additional study of the genus may reveal whether this is due to cyanogenesis or another mechanism. Because HCN is a general toxin affecting oxidative respiration, the potential of *C*.sp_P as a biocontrol agent may be limited. However, further work to develop methods to avoid off-target effects and target mosquito larvae specifically (*e*.*g*. through use of larval attractants or through treatment of breeding sites) is warranted.

## Methods

### Ethics statement

This study was carried out in strict accordance with the recommendations in the Guide for the Care and Use of Laboratory Animals of the National Institutes of Health. The protocol was approved by the Animal Care and Use Committee of the Johns Hopkins University (permit number MO15H144).

### Mosquito strain maintenance

*Anopheles gambiae* Keele strain mosquitoes were reared at 27 °C and 80% residual humidity with a 14/10 hour light/dark cycle. Adult females were fed on anaesthetized mice for egg collection, larvae were reared on TetraMin^®^ fish food and cat food pellets, and adults were provided 10% sucrose *ad libitum*.

### Bacterial culture and growth curve measurements

All bacterial stocks were maintained in 25% glycerol stocks at −80 °C. To generate these stocks, liquid LB media was inoculated with a single bacterial colony and grown overnight at 30 °C with constant shaking. Cultures were pelleted and washed two times with 1× PBS and then mixed 1:1 with sterile 50% glycerol solution and stored at −80 °C. To culture *Chromobacterium* species Panama (*C*. sp_P), *Comamonas sp*.^10^, *C*. *violaceum* (ATCC 12472)^25^, *C*. *aquaticum* (DSM 19852)^67^, *C*. *vaccinii* (DSM 25150)^68^, and *C*. *subtsugae* (DSM 17043)^23^ for experiments, 1 µL of pure bacterial freezer stock was added to 5 mL LB and grown at 30 °C with shaking for 16–18 hours. For most larval survival experiments, overnight liquid culture was washed twice with sterile 1X PBS and resuspended in additional 1X PBS to 1.0 (±0.1) OD_600._ For the experiment testing multiple species of *Chromobacterium* in parallel, overnight liquid cultures were washed twice with 1X PBS. *C*. *subtsugae* overnight cultures routinely have lower CFU/mL than the other species, therefore *C*. *subtsugae* was further concentrated 5X and the other cultures were used without concentration. This step was performed in an attempt to standardize CFU/mL within an order of magnitude for all species. 100uL of each culture were then added to 5 mL larval breeding water, resulting in an approximate bacterial density of 10^7^ CFU/5 mL larval water for all species except *C*. *subtsugae*, for which it was approximately 10^8^ CFU/5 mL larval water. Liquid cultures were serially diluted and grown on LB agar to estimate CFU/mL at the time of treatment. For growth curve measurements, 10 mL of an overnight *C*.sp_P culture was used to inoculate 1 L liquid LB in triplicate. Cultures were then grown for eight hours at 30 °C with shaking and OD_600_ was measured every 1–1.5 hours during the incubation.

### Larval survival after exposure to live C.sp_P or C.sp_P-treated breeding water

Larvae for all experiments were a mix of 1^st^ and 2^nd^ instars. For experiments using live bacteria, ten *An*. *gambiae* larvae were placed in individual wells of a 6-well cell culture dish containing 5 mL water and approximately 3 mg ground larval food (unless stated otherwise). Ground larval food was used for all larval experiments, and consisted of liver powder, tropical fish flake food, and rabbit food pellets mixed in a 2:1:1 ratio. 100uL of prepared bacterial culture was added to the larval water and survival was monitored for four days. For the experiment measuring larval survival and HCN production in concert, ten *An*. *gambiae* larvae were placed in 18 separate 150 mm petri dishes containing 75 mL water and approximately 45 mg ground larval food. To three petri dishes, 500 µL 1X PBS was added. To fifteen petri dishes, 500 µL 1.0 OD_600_ *C*.sp_P bacterial culture was added. At 4, 6.5, 7.5, 24, and 48 hours after treatment, all 75 mL was harvested from three of the fifteen *C*.sp_P-treated petri dishes and used for HCN analysis. At 3, 4, 5, 6, 6.5, 7.5, 24, and 48 hours after treatment, survival from all remaining petri dishes was recorded.

### Larval survival after exposure to filtered (i.e. cell-free) C.sp_P-treated water

Larvae were treated with *C*.sp_P or PBS as described above. After death (6–8 hours post exposure), larval water from PBS and *C*.sp_P -treated wells was vortexed, centrifuged at 5000 rpm for 3 minutes, and passed through a 0.2 µm syringe filter, which removes live bacterial cells. Filtrate sterility was confirmed in two ways: in an initial experiment, filtrate was spread on LB agar and was confirmed to yield zero colonies after multiple days of growth. In subsequent experiments, filtrate was left at room temperature for multiple days and was confirmed to never become turbid after filtration. Any additional treatments to the water were performed at this point (*e*.*g*. size-fractionation, heating, vacuum centrifugation) and 1 mL aliquots of the filtered water were then used to treat pools of 5 *An*. *gambiae* larvae (1^st^ and 2^nd^ instars) in 24-well cell culture dishes. No food was added, and survival was recorded after an overnight (16–17 hours) or 24-hour incubation. When heating the filtered water, 1 mL aliquots were placed in a heat block in sealed 1.5 mL microcentrifuge tubes for one hour. Samples were mixed by hand and allowed to return to room temperature before use.

### Size fractionation of filtered larval breeding water

To size-fractionate filtered PBS and *C*.sp_P-treated larval water, an equal volume of each was loaded onto an Amicon Ultra-4 Centrifugal Filter Unit with Ultracel-10 membrane and spun at 7917 × g for 15 minutes. The filtrate was then loaded onto an Amicon Ultra-4 Centrifugal Filter Unit with Ultracel-3 membrane and spun at 7917 × g for 25 minutes. The concentrate from the Ultracel-10 filtration unit contains molecules greater than 10 kDa, while the concentrate from the Ultracel-3 filtration unit contains molecules less than 10 kDa but greater than 3 kDa. The final filtrate contains molecules less than 3 kDa. Sterile water was added to both concentrates to raise their volumes to equal that of the final filtrate.

### Vacuum centrifugation of filtered larval breeding water

*C*.sp_P- and PBS-treated larval water that had been previously isolated (for size fractionation experiments and stored at 4 °C) was used for survival analysis after vacuum centrifugation. *C*.sp_P-treated water used for HCN quantification after vacuum centrifugation was generated fresh as described above. Aliquots of these water samples were vacuum centrifuged in open 1.5 mL microcentrifuge tubes for 30 minutes at room temperature. Control aliquots were incubated at room temperature in sealed microcentrifuge tubes during centrifugation. Sterile water was added to centrifuged samples to compensate for loss of volume.

### Hydroxocobalamin treatment of filtered larval breeding water

*C*.sp_P- and PBS-treated larval water used for this experiment was isolated and filtered on two independent days as described above. Larvae were treated with these water samples twice, once on the day they were isolated and then on a later date. In the time between treatment days, water samples were stored at 4 °C. 1 mL of filtered *C*.sp_P- or PBS-treated water was applied to pools of 5 *An*. *gambiae* larvae, and to half the pools from each treatment, filter sterilized OHCbl in water was added to the larval water at a final concentration of 0.2 mg/mL. Filter sterilized water was added to the remaining pools as a control.

### Treatment of larvae with NaCN

An 18 mg/mL stock solution of NaCN dissolved in water was serially diluted 1:5, 1:10, 1:20, 1:40, and 1:80, resulting in NaCN concentrations of 3.6, 1.8, 0.9, 0.45, and 0.23 mg/mL, respectively. One milliliter of each dilution (plus water as a negative control) was applied to five *An*. *gambiae* larvae (a mixture of 1^st^ and 2^nd^ instars) in three wells of a 24-well cell culture plate. No larval food was added. After 24 hours, mortality in each well was recorded.

### Hydrogen cyanide measurements

For experiments measuring HCN concentration in bacterial culture, in NaCN solution, and after vacuum centrifugation, HCN was measured using the Hach Cyanide Test Kit CYN-3 (Lot A5316) according to the manufacturer’s instructions. For the experiment measuring larval survival and HCN produced by *C*.sp_P in larval breeding water over time, EPA Method 335.4 was used. This method utilizes semi-automated colorimetry to assess total cyanide and was performed by SGS Accutest (Dayton, NJ). Both methods were used to measure HCN concentration in the same NaCN stock, and the result using the Hach Cyanide Test Kit was 30.75 mg/L, while that from semi-automated colorimetry was 18 mg/L. Semi-automated colorimetry is more sensitive and is less subject to user error than the Hach Cyanide Test Kit, and the different results from the two methods indicate that the Hach test kit overestimated cyanide concentrations by a factor of 1.7083. The following correction was therefore applied to all data obtained using the Hach Kit: *y* = *x* * 1/1.7083 where $y$ = corrected HCN value and $x$ = original HCN value.

### Larval development and survival after non-lethal exposure to C.sp_P

A mix of first and second instar *An*. *gambiae* larvae were treated with *C*.sp_P or 1X PBS (negative control) in groups of 350 in 175 mL of water containing 105 mg ground larval food. *C*.sp_P overnight liquid culture was washed 2X with 1X PBS and approximately 3.6 × 10^6^ CFU (rep 1: 2.4 × 10^6^, rep 2: 4.8 × 10^6^) of *C*.sp_P were added to the larval breeding water. After a two-day exposure, larval survival was recorded and *C*.sp_P- and PBS-treated larvae were rinsed twice with distilled water and transferred to large larval pans containing 2 L distilled water supplemented with 2 pellets of cat food and 15 mg of TetraMin^®^ fish food. Every 2–3 days, an additional 15 mg of fish food was added. Pupation events were recorded daily beginning 3 days post exposure and continuing until all pupae eclosed or died. Larval cadavers were removed daily from larval pans and the total number of larvae that either died as larvae or were still alive (but never pupated) was recorded at the conclusion of the experiment.

### Statistical analysis

Survival over time was assessed using Cox proportional hazards models with treatment, experimental replicate, and replicate pool of larvae as predictor variables in the model. Models were fit using “coxph” in the Survival package in R^69^. Survival at a single time point was analyzed using binomial logistic regression with treatment(s), experimental replicate, replicate pool of larvae, and date of larval water harvest as predictor variables where appropriate. Summary results and p-values from each model can be found in Table S1. Binomial logistic regressions were performed in R using “glm” in the stats package, and pairwise comparisons were conducted using “glht” in the multcomp package. Rate of eclosion was assessed using a log rank test in R implemented with “survdiff” in the survival package.

### Data Availability Statement

Raw data for all experiments can be found in the Supplementary Dataset.

## Electronic supplementary material

Supplementary Information

Supplementary Dataset 1
