# Supplementary material for: Hydrogen cyanide produced by the soil bacterium Chromobacterium sp. Panama contributes to mortality in Anopheles gambiae mosquito larvae
Source: Sci Rep. 2018 May 29;8:8358. doi: 10.1038/s41598-018-26680-2 (PMC5974309; doi:10.1038/s41598-018-26680-2)
Supplement: Supplementary file 1 — Supplementary Information [file 41598_2018_26680_MOESM1_ESM.pdf]

Supplementary Information for:

Hydrogen cyanide produced by the soil bacterium Chromobacterium sp. Panama contributes to mortality in Anopheles gambiae mosquito larvae

Sarah M. Short, Sarah van Tol, Hannah J. MacLeod, George Dimopoulos

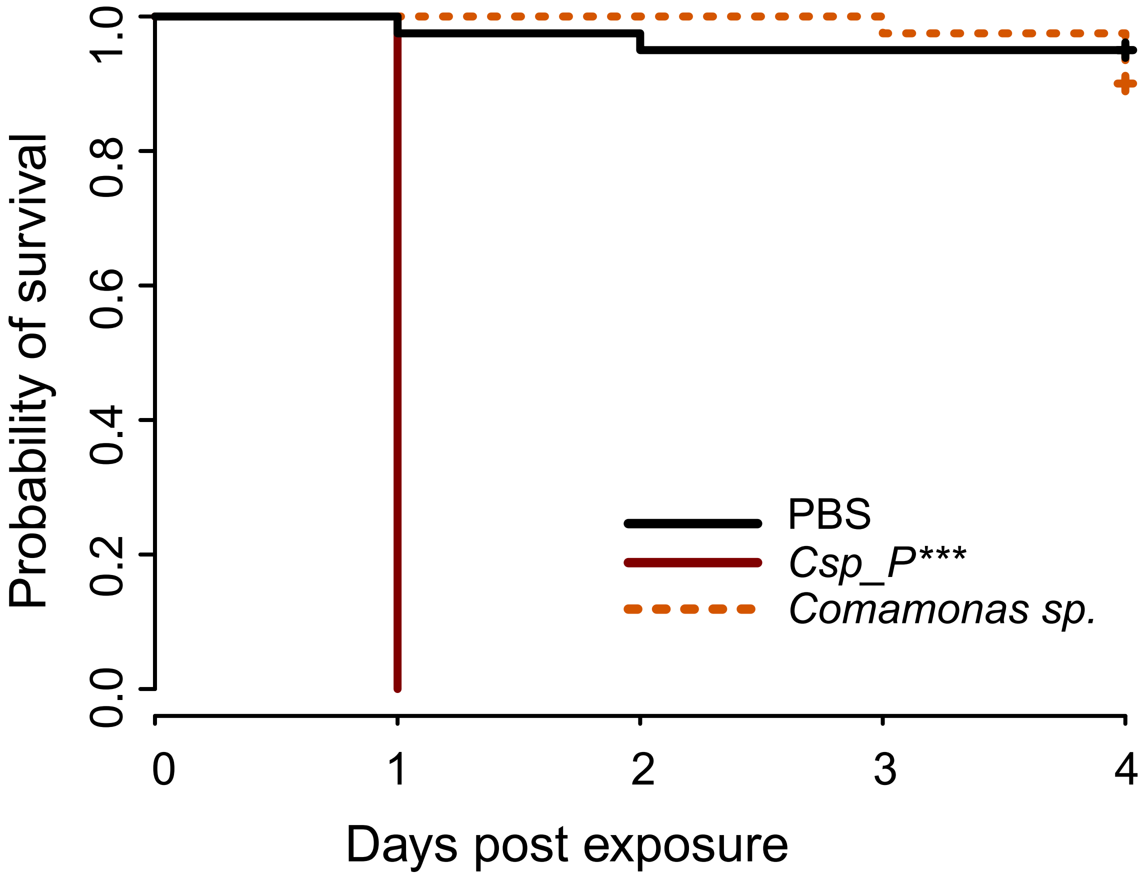

Figure S1: Exposure to *Comamonas sp.*, a common mosquito midgut bacterium does not induce larval mortality. First and second instar *An. gambiae* larvae were treated in groups of ten in 5mL of water containing 10mg of ground larval food. *C.sp_P* or *Comamonas sp.*overnight cultures were diluted to 1.0 (±0.1) OD_{600} and 100µL of each culture was added to the larval breeding water. 100µL of 1X PBS was added as a control and survival of larvae was monitored for four days. Compared to the 1X PBS control, *C.sp_P* treated larvae had significantly lower survival ($p = 2.14 \times 10^{-7}$) while *Comamonas sp.* treated larvae did not ($p = 0.435$). The experiment was repeated two independent times and for each experimental replicate two pools of ten larvae were measured per treatment. Data were analyzed using a Cox proportional hazards model. Total sample sizes are as follows: PBS = 40, *C.sp_P*= 40, *Comamonas sp.* = 40.

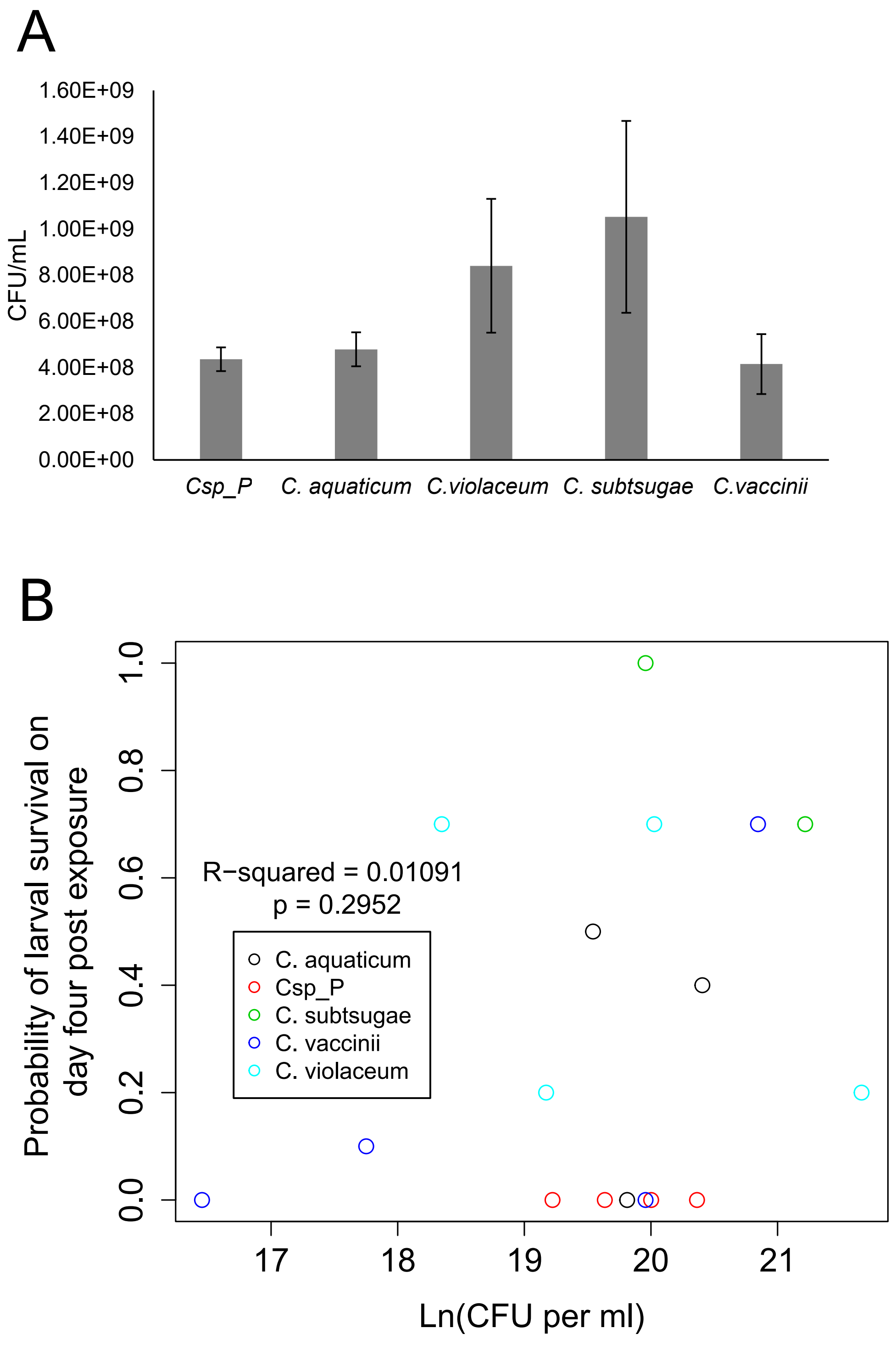

Figure S2: (A) CFU added to larval breeding water for each species of *Chromobacterium*. Live bacteria were added at the above average concentrations to 5mL larval breeding water over three replicate experiments. **(B) CFU across all species of *Chromobacterium* does not predict larval survival.** For the five species of *Chromobacterium* used to treat *An. gambiae* larvae, we performed a linear regression to assess the relationship between CFUs added to the larval water and larval mortality. We found no correlation between these factors (r-squared = 0.01091, p = 0.2952).

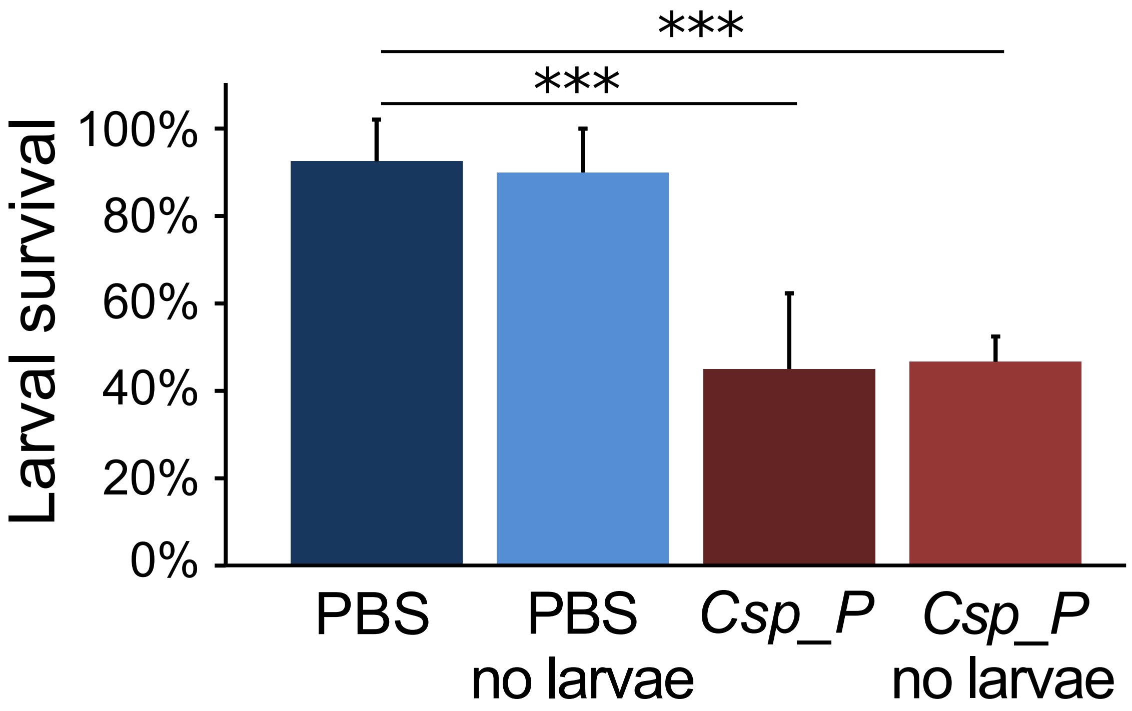

Figure S3: Larvicidal activity not dependent on the presence of larvae. Larval breeding water was treated with either 1X PBS or *C.sp_P*, and incubated for 6-8 hours until all larvae in the *C.sp_P* treatment were dead. In half the treatments, no larvae were added to the larval breeding water. Larval water was then collected and filtered using a 0.2µm filter to remove all live bacterial cells. This filtered water was then used to treat new pools of five *An. gambiae*larvae and survival was recorded after 16-17 hours exposure to the filtered water. The "PBS" and "*C.sp_P*" data presented here are the same as those presented in Figure 3. Larvae exposed to *C.sp_P* treated water suffered significant mortality regardless of whether larvae were co-incubated with *C.sp_P* bacteria before filtration (with larvae, $p = 0.0011$, without larvae, $p = 0.00097$). The graph shows average percent survival across replicate experiments and error bars represent one standard deviation. For each experiment two pools of five larvae were measured per treatment and the experiment was repeated three independent times. For all treatments, total sample size was $n = 30$.

Table S1: Model outputs for Statistical Analyses

Figure 1A:

```
coxph(formula = Surv(day, status, type = c("right")) ~ treatment + rep + pool)
```

n= 355, number of events= 97

	coef	exp(coef)	se(coef)	z	Pr(>|z|)	
CspP 10^5	0.69003	1.99378	0.41689	1.655	0.0979	.
CspP 10^3	-0.45264	0.63595	0.52716	-0.859	0.3905	
CspP 10^1	-0.65412	0.51990	0.55782	-1.173	0.2409	
CspP 0.1	-1.58830	0.20427	0.78183	-2.032	0.0422	*
CspP 10^7	3.95136	52.00587	0.42587	9.278	<2e-16	***
rep2	0.47306	1.60490	0.26506	1.785	0.0743	.
rep3	0.26788	1.30719	0.27574	0.971	0.3313	
pool2	0.05954	1.06135	0.20336	0.293	0.7697	

Figure 1B:

```
coxph(formula = Surv(day, status, type = c("right")) ~ treatment + rep + pool)
```

n= 240, number of events= 102

	coef	exp(coef)	se(coef)	z	Pr(>|z|)	
CspP 10^5	-0.33149	0.71785	0.39705	-0.835	0.404	
CspP 10^3	0.36408	1.43919	0.34165	1.066	0.287	
CspP 10^1	0.15112	1.16314	0.35436	0.426	0.670	
CspP 0.1	-0.02647	0.97388	0.37166	-0.071	0.943	
CspP 10^7	1.35025	3.85838	0.32948	4.098	4.17e-05	***
rep2	-0.03130	0.96918	0.20013	-0.156	0.876	
pool2	-0.02145	0.97878	0.20064	-0.107	0.915	

Figure 2:

```
coxph(formula = Surv(day, status, type = c("right")) ~ treatment + rep)
```

n= 260, number of events= 141

	coef	exp(coef)	se(coef)	z	Pr(>|z|)	
C. aquaticum	1.73922	5.69291	0.31997	5.436	5.46e-08	***
CspP	3.01887	20.46817	0.31796	9.494	< 2e-16	***
C. subtsugae	-0.12424	0.88317	0.64027	-0.194	0.846	
C. vaccinii	2.32464	10.22296	0.31987	7.268	3.66e-13	***
C. violaceum	1.36668	3.92232	0.33550	4.074	4.63e-05	***
rep2	0.04868	1.04988	0.23295	0.209	0.834	
rep3	0.09991	1.10507	0.23457	0.426	0.670	
rep4	-0.45661	0.63343	0.25110	-1.818	0.069	.

Figure 3:

```
glm(formula = status ~ rep + treat/pool, family = binomial(link = logit))
```

	Estimate	Std. Error	z value	Pr(>|z|)	
(Intercept)	-2.9892	0.9428	-3.170	0.00152	**
rep2	0.8844	0.8837	1.001	0.31697	
rep3	-0.1025	0.8416	-0.122	0.90303	
treatCspP	3.6174	0.8678	4.169	3.06e-05	***
treatPBS:poo2	-17.0511	2364.3872	-0.007	0.99425	
treatCspP:pool2	2.0318	1.1475	1.771	0.07662	.

Figure 4A:

```
glm(formula = status ~ rep + treat, family = binomial(link = logit))
```

	Estimate	Std. Error	z value	Pr(>|z|)	
(Intercept)	-2.7030	0.6940	-3.895	9.82e-05	***
rep2	0.4258	0.5328	0.799	0.424	
rep3	0.1479	0.5382	0.275	0.784	
rep4	0.1479	0.5382	0.275	0.784	
treatcsp	2.7246	0.6808	4.002	6.27e-05	***
treatcsp10k	-16.0520	1029.4223	-0.016	0.988	
treatcsp3-10k	-1.1525	1.1778	-0.979	0.328	
treatcsp3k	2.7246	0.6808	4.002	6.27e-05	***

treatpbs10k	-0.4328	0.9423	-0.459	0.646
treatpbs3-10k	-0.4328	0.9423	-0.459	0.646
treatpbs3k	-1.1736	1.1776	-0.997	0.319

Figure 4B:

Heat treatment experiment:

Room temperature:

```
glm(status~rep+treatment/well,data=rt, family=binomial(link=logit))
```

	Df	Deviance	Resid. Df	Resid. Dev	Pr(>Chi)	
NULL			59	74.920		
rep	2	2.9435	57	71.976	0.2295194	
treatment	1	14.8347	56	57.142	0.0001174	***
treatment:well	2	0.9822	54	56.159	0.6119638	

37 degrees:

```
glm(status~rep+treatment/pool,data=ts, family=binomial(link=logit))
```

	Df	Deviance	Resid. Df	Resid. Dev	Pr(>Chi)	
NULL			59	73.304		
rep	2	1.4919	57	71.812	0.474286	
treatment	1	8.5075	56	63.304	0.003537	**
treatment:pool	2	3.4841	54	59.820	0.175158	

55 degrees:

```
glm(status~rep+treatment/pool,data=ff, family=binomial(link=logit))
```

	Df	Deviance	Resid. Df	Resid. Dev	Pr(>Chi)	
NULL			59	73.304		
rep	2	1.3964	57	71.907	0.49747	
treatment	1	24.2913	56	47.616	8.281e-07	***
treatment:pool	2	5.1472	54	42.469	0.07626	.

70 degrees:

```
glm(status~rep+treatment/pool,data=s, family=binomial(link=logit))
```

	Df	Deviance	Resid. Df	Resid. Dev	Pr(>Chi)	
NULL			59	81.503		
rep	2	0.9590	57	80.544	0.6191014	
treatment	1	12.2854	56	68.259	0.0004565	***
treatment:pool	2	2.1969	54	66.062	0.3333882	

90 degrees:

```
glm(status~rep+treatment/pool,data=n, family=binomial(link=logit))
```

	Df	Deviance	Resid. Df	Resid. Dev	Pr(>Chi)	
NULL			59	73.304		
rep	2	1.933	57	71.371	0.38049	
treatment	1	34.443	56	36.929	4.39e-09	***
treatment:pool	2	5.951	54	30.977	0.05101	.

Figure 4C:

Initial model:

```
glm(status~rep+waterharvest+treat*vac_cent/pool,data=og, family=binomial(link=logit))
```

Final model:

```
glm(status~rep+treat*vac_cent,data=og, family=binomial(link=logit))
```

Anova:

	Df	Deviance	Resid. Df	Resid. Dev	Pr(>Chi)	
NULL			238	313.42		
rep	1	11.087	237	302.33	0.0008692	***
treat	1	23.458	236	278.88	1.277e-06	***
vac_cent	1	15.764	235	263.11	7.174e-05	***
treat:vac_cent	1	27.467	234	235.65	1.598e-07	***

Figure 6:

Original model:

```
glm(status~waterharvest+exptrep+bact*antidote/pool, family=binomial(link=logit))
```

Final model:

```
glm(status~bact*antidote, family=binomial(link=logit))
```

	Df	Deviance	Resid. Df	Resid. Dev	Pr(>Chi)	
NULL			159	154.43		
bact	1	4.1680	158	150.26	0.0411951	*
antidote	1	14.4453	157	135.81	0.0001443	***
bact:antidote	1	5.0559	156	130.76	0.0245421	*

Figure 7A:

```
survdiff(formula = Surv(day, status) ~ treatment)
```

	N	Observed	Expected	(O-E)^2/E	(O-E)^2/V
treatment=csp	262	140	159	2.38	5.78
treatment=pbs	476	377	358	1.06	5.78

Chisq= 5.8 on 1 degrees of freedom, p= 0.0162
